# Autoinfarction of the Parathyroid Gland: An Unusual Presentation of Primary Hyperparathyroidism

**DOI:** 10.1155/crie/2683073

**Published:** 2026-06-29

**Authors:** Jaskaran Batra, Tanya Aggarwal, Akshaya Ramachandran, Amy Neumeister, Laura Graeff-Armas

**Affiliations:** ^1^ Division of Diabetes, Endocrinology and Metabolism, University of Nebraska Medical Center, Omaha, Nebraska, USA, unmc.edu

## Abstract

**Background:**

Primary hyperparathyroidism (PHPT) is commonly caused by a parathyroid adenoma and typically presents with hypercalcemia and elevated parathyroid hormone (PTH) levels. Spontaneous infarction of parathyroid adenoma is a rare phenomenon that can lead to spontaneous resolution of hypercalcemia.

**Case Presentation:**

We report the case of a 48‐year‐old male with a history of PHPT diagnosed 7 years prior and was lost to follow up, who presented with left‐sided facial and finger paresthesia. Initial labs revealed low ionized calcium with a normal PTH level. Imaging identified a heterogeneous mass adjacent to the left thyroid lobe. Biopsy confirmed infarcted parathyroid adenoma. The patient’s prior PHPT had spontaneously resolved, but he now presented with symptomatic hypocalcemia likely due to adenoma infarction.

**Discussion:**

Spontaneous infarction of a parathyroid adenoma is an uncommon cause of remission in PHPT. While transient remission of hypercalcemia has been documented, long‐term outcomes remain variable. Our case is unusual as the patient presented with symptomatic hypocalcemia years after initial diagnosis.

**Conclusion:**

Autoinfarction of parathyroid adenomas can result in spontaneous remission of PHPT but may also lead to unexpected complications such as hypocalcemia. Ongoing follow‐up is essential to monitor both recurrence of hypercalcemia and the development of hypocalcemia.

## 1. Introduction

Primary hyperparathyroidism (PHPT) classically presents with elevated calcium levels in the setting of high or inappropriately normal parathyroid hormone levels with the most common etiology being parathyroid adenoma. In asymptomatic individuals who are less than 50 years of age or those with symptomatic hypercalcemia, the American Association of Clinical Endocrinology recommends parathyroidectomy as the definitive treatment. In rare cases, adenoma can undergo spontaneous infarction, thereby leading to the resolution of hypercalcemia. We present a case of hypercalcemia secondary to PHPT which resolved initially and subsequently developed symptomatic hypocalcemia deemed to be secondary to an infarcted parathyroid adenoma after spontaneous hemorrhage.

## 2. Case Report

A 48‐year‐old male with medical history of hypertension presented to the emergency department (ED) with complaints of sore throat, left sided facial numbness and tingling, along with numbness and tingling of his left fingers for 1 day. The physical exam was nonfocal with no obvious weakness in upper or lower extremities and normal cranial nerve examination.

Initial lab work revealed normal complete blood count and basic metabolic panel; however, his calcium was 9.8 mg/dL (8.6–10.4) with low ionized calcium of 1.12 mmol/L (1.18–1.30), normal parathyroid hormone of 54 pg/mL (12–88) and a low 25(OH)D level of 26 ng/mL (30–80). On further chart review he was diagnosed with PHPT 7 years ago and was recommended surgery; however, he was lost to follow‐up. Labs performed at that time were relevant for parathyroid hormone (PTH) of 125 pg/mL, total calcium of 11.2 mg/dL, 24 h urine calcium of 507 mg/day (100–300), creatinine of 2083 mg (500–2000), and phosphorus of 2.5 mg/dL (2.4–4.5).

**Figure 1:**
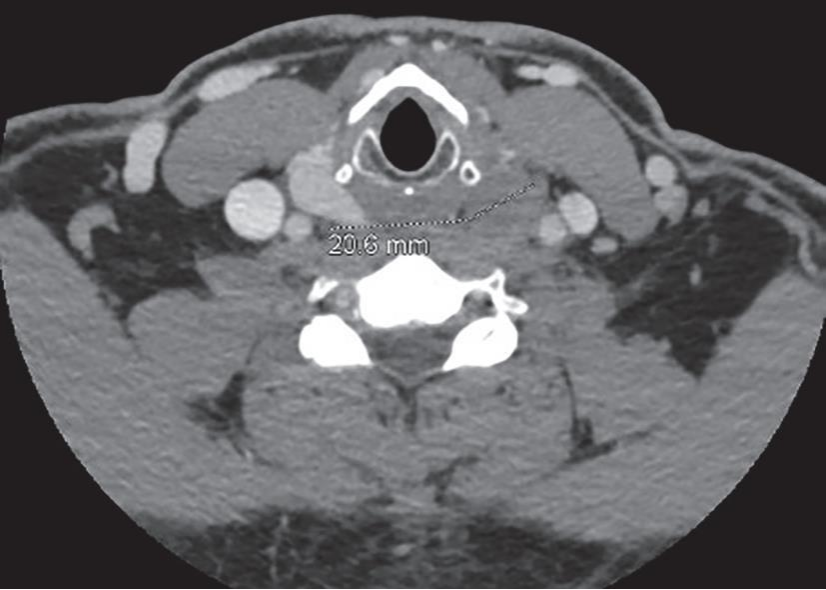
CT of the neck showing mass‐like fullness in the left compartment of the neck.

**Figure 2:**
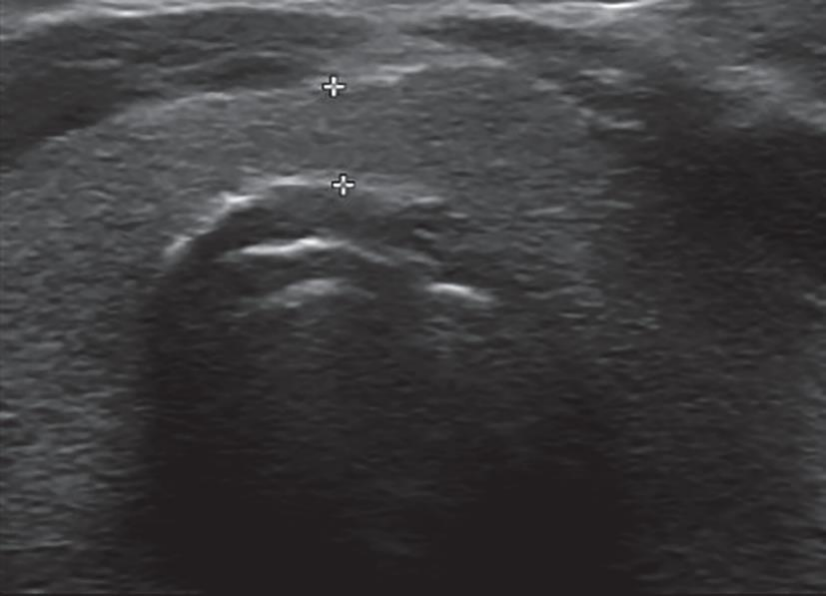
Ultrasound of the neck in transverse view showing a left sided hypoechoic nodule.

**Figure 3:**
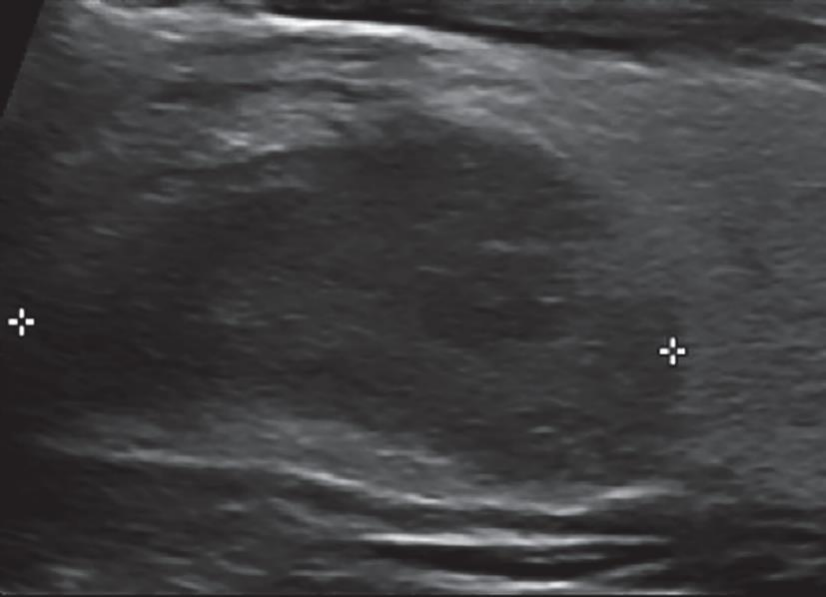
Ultrasound of the neck in longitudinal view showed a 3.3 cm heterogenous mildly hypoechoic avascular nodule abutting the posterior aspect of the left superior thyroid lobe.

Computed tomography (CT) of head and neck performed in the ED because of concern for a stroke which showed a fullness in the left visceral compartment of the neck concerning for a possible lateral pharyngoesophageal Killian–Jameson diverticulum with inflammation and secondary effusion (Figure 1). Ultrasound neck showed a 3.3 cm heterogenous mildly hypoechoic avascular nodule abutting the posterior aspect of the left superior thyroid lobe with possible differentials being an abscess, mucosal diverticulum with debris or a hypovascular solid lesion such as parathyroid neoplasm or exophytic thyroid nodule (Figures 2, 3).

ENT was consulted and recommended an esophagogram to rule out diverticulum and an MRI of the neck. The esophagogram was normal, and MRI showed a 3.5 cm lesion abutting the posterior superior aspect of the left thyroid lobe with possible differentials being both benign and malignant thyroid and parathyroid lesions, inflamed esophageal diverticulum, abscess and less likely branchial cleft anomaly. A 4D CT scan of the neck with contrast was subsequently done which did not demonstrate the enhancement characteristics of a parathyroid adenoma.

**Figure 4:**
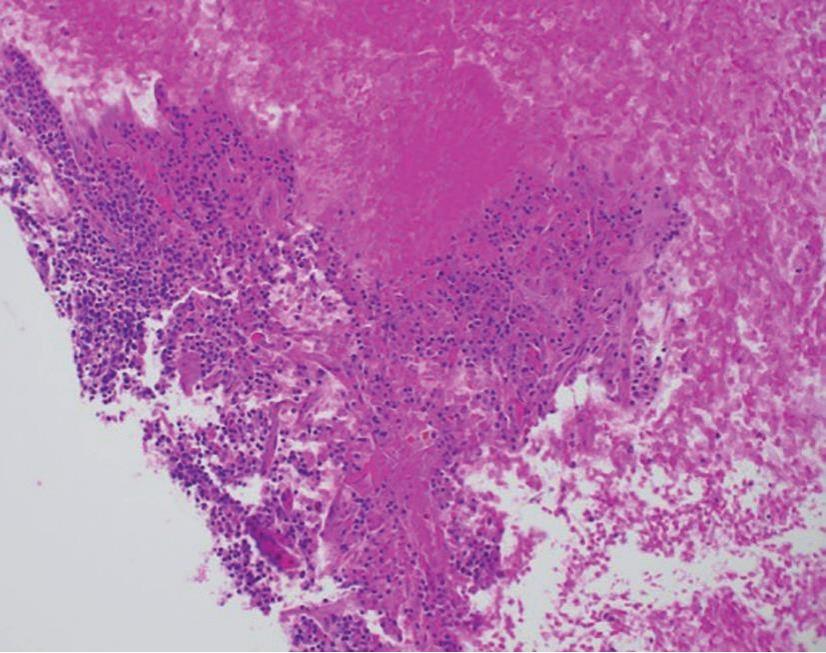
Biopsy showed hemorrhage and fibrosis of the parathyroid gland.

**Figure 5:**
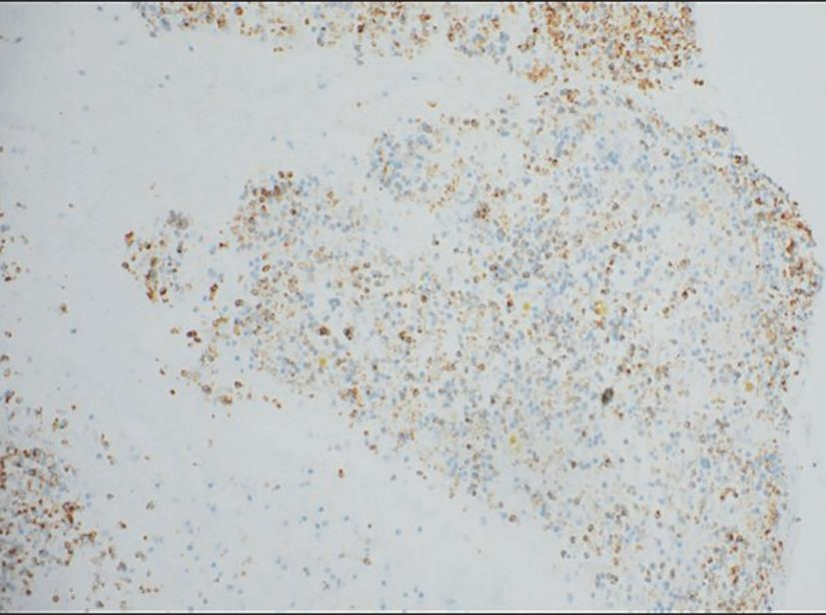
Cells staining positive for PTH.

He underwent a biopsy of the mass whose pathology demonstrated cellular parathyroid tissue fragments with nests of chief cells with intervening adipose tissue along with hemorrhagic debris and focal fibrosis consistent with infarction of parathyroid adenoma (Figures 4, 5). The patient was given supportive treatment and one dose of IV calcium after which the patient’s symptoms resolved, and his calcium levels normalized. He was discharged without any calcium or vit D supplements. Repeat CT of the neck after 6 months still showed persistence of the neck mass, but it had decreased in size, with normal serum calcium so he chose the option of watchful waiting with periodic monitoring of his calcium levels.

## 3. Discussion

Spontaneous infarction of parathyroid adenoma is an uncommon event that has been described previously by Kovacs and Gay [1]. They postulated that the large size of the adenomas could predispose to infarction as they outgrew their blood supply. They presented a series of 12 patients who had a spontaneous infarction of their parathyroid gland, 7/12 patients had a normal calcium level, 3/12 had a low level, and 2/12 had a high‐level postinfarction. The low calcium could be because of abrupt cessation of PTH secretion after infarction along with possible hungry bone syndrome while high calcium could be because of release of preformed PTH from necrotic cells after infarction. The calcium levels can fluctuate depending upon the time after the infarct that the calcium is checked. Necrosis of the parathyroid gland may manifest with pain, swelling, tenderness in the neck, along with hoarseness and dysphagia, and rarely ecchymosis of the anterior neck. Nylen et al. [2] proposed the term parathyroid apoplexy and classified it into three types—necrosis without hemorrhage, necrosis with intracapsular hemorrhage, and necrosis with extracapsular hemorrhage. Kozlow et al. [3] summarized 16 cases of extracapsular hemorrhage, 11 out of which occurred in preexisting adenomas, three within parathyroid hyperplasia, and two within parathyroid cysts. Wooten and Orzeck [4] summarized all the three categories of parathyroid infarction and they calculated the mean PTH change which was 69% and postulated that postinfarction resolution of hypercalcemia may be indicated by a higher PTH drop given that postsurgery a 50% fall in 20 min is a marker of successful resection.

Literature does support short term remission of hypercalcemia due to autoinfarction of parathyroid adenoma, but there is a risk of recurrence of hypercalcemia which warrants periodic laboratory monitoring postremission [5]. In a 10‐year prospective study by Silverberg et al. [6] about 27% of people with asymptomatic hyperparathyroidism had progression of their disease requiring surgery. In our case, the patient had a history of asymptomatic PHPT 7 years prior to his current presentation with symptomatic hypocalcemia. While this is unusual and has not been commonly reported, it sheds light on the importance of appropriate follow‐up and monitoring of PHPT cases for recurrent hypercalcemia as well as hypocalcemia. We think that the abrupt drop in serum calcium in our patient caused the low ionized calcium and normal total calcium which led to his symptoms. The CARE checklist for this case report has been provided in the Supporting Information.

## 4. Conclusion

Spontaneous infarction of parathyroid adenoma leading to remission of PHPT is rare. However, the risk of recurrent hypercalcemia and hypocalcemia is well reported. Given the unpredictable nature of the disease course of postinfarction, it is vital that these patients be monitored at appropriate intervals to avoid complications from hypocalcemia.

## Author Contributions

Jaskaran Batra, Tanya Aggarwal, and Akshaya Ramachandran were involved in data acquisition, literature review, and drafting of the manuscript. Laura Graeff‐Armas and Amy Neumeister assisted in drafting and revising the manuscript. Jaskaran Batra was responsible for the clinical management of the patient. Laura Graeff‐Armas also contributed to study conception, supervision, and critical revision of the manuscript for important intellectual content.

## Funding

No funding was received for this manuscript.

## Consent

Written informed consent was obtained from the patient for publication of this case report and any accompanying images.

## Conflicts of Interest

The authors declare no conflicts of interest.

## Supporting Information

Additional supporting information can be found online in the Supporting Information section.

## Supporting information


**Supporting Information** The CARE checklist for this case report is provided as supporting information.

## Data Availability

Data sharing is not applicable to this article as no datasets were generated or analyzed during the current study.
